# A flexible module-based cognitive behavioral therapy for gaming disorder: study protocol of a randomized controlled trial

**DOI:** 10.1186/s13063-025-09147-4

**Published:** 2025-10-02

**Authors:** Elin Arvidson, Annika Hofstedt, Anna Söderpalm Gordh

**Affiliations:** 1 Clinic for Gambling Addicition and Screen Health, Department of Addiction and Dependency, Sahlgrenska University Hospital, Gothenburg, Sweden; 2 Sahlgrenska Academy, Institution for Neuroscience and Physiology, Section for Psychiatry and Neurochemistry, University of Gothenburg, Gothenburg, Sweden

**Keywords:** Gaming disorder (GD), Psychiatry, Treatment, Cognitive behavioral therapy (CBT), Randomized controlled trial (RCT), Behavioral addiction, Adults, Adolescents

## Abstract

**Background:**

Knowledge about gaming disorder has increased in recent years. However, more research is needed regarding treatment options for gaming disorder. The most studied treatment option today is cognitive behavioral therapy (CBT). However, due to the low number of high-quality trials, the certainty in the evidence is low. Only a handful of previous studies are randomized controlled trials, further affecting the evidence level. Many previously studied treatments are also resource-intensive, making it difficult to implement in routine care. In addition, few treatment studies so far include adults. Therefore, we aim to evaluate a flexible manual-based CBT-program for gaming disorder in a randomized controlled trial, including both adults and adolescents.

**Methods:**

A total of 160 patients will be recruited. Participants will be randomized to an intervention group receiving CBT treatment for approximately 12 weeks or to a waitlist control group. The primary outcome measure is the score on the Internet Gaming Disorder Scale 9- Short form. The questionnaire measures severity of gaming disorder before and after treatment. Assessments will be made at five time points, from the first visit to a 3-month follow-up. The participants randomized to the control group will remain on a waitlist for 12 weeks. During this time, the participants in the control group will receive the same questionnaires at the same time points as the intervention group. After 12 weeks, the control group will be offered the same treatment as participants in the intervention group. In an additional study, all participants will be followed up until 24 months after treatment.

**Discussion:**

This trial will evaluate the effects of a 12-week CBT-treatment for patients with gaming disorder, compared to no treatment. Increasing the knowledge of the effectiveness of CBT for gaming disorder will improve the ability to offer evidence-based care for this group of patients.

**Trial registration:**

Clinicaltrials.gov NCT05328596. Registered on April 22, 2022.

## Administrative information

Note: the numbers in curly brackets in this protocol refer to SPIRIT checklist item numbers. The order of the items has been modified to group similar items (see http://www.equator-network.org/reporting-guidelines/spirit-2013-statement-defining-standard-protocol-items-for-clinical-trials/).

**Table:** 

Title {1}	The effectiveness of flexible module-based cognitive behavioral therapy for gaming disorder in an adult population: study protocol of a randomized controlled trial
Trial registration {2a and 2b}.	Registered at ClinicalTrials.gov, ID: NCT05328596. April 22, 2022
Protocol version {3}	Protocol version 1, August 1, 2025
Funding {4}	The study was supported by Svenska Spel’s Independent Research Council Grant number FO2021-0007, the Swedish Research Council (2023–00202) and the Swedish government and county councils (ALF Grants) number ALFGBG-983591.
Author details {5a}	Elin Arvidson*: Clinic for Gambling Addiction and Screen Health, Department of Addiction and Dependency, Sahlgrenska University Hospital, Gothenburg, Sweden *Annika Hofstedt:* Clinic for Gambling Addiction and Screen Health, Department of Addiction and Dependency, Sahlgrenska University Hospital, Gothenburg, Sweden and the Sahlgrenska Academy, Institution for Neuroscience and Physiology, Section for Psychiatry and Neurochemistry, University of Gothenburg, Gothenburg, Sweden. *Anna Söderpalm Gordh*: Clinic for Gambling Addiction and Screen Health, Department of Addiction and Dependency, Sahlgrenska University Hospital, Gothenburg, Sweden and the Sahlgrenska Academy, Institution for Neuroscience and Physiology, Section for Psychiatry and Neurochemistry, University of Gothenburg, Gothenburg, Sweden. Gothenburg, Sweden.
Name and contact information for the trial sponsor {5b}	Department of Addiction and Dependency, Sahlgrenska University Hospital, Gothenburg, Sweden.
Role of sponsor {5c}	The sponsor and funder had no influence on the study design, writing or decision to submit the report. The principal investigators had all responsibility for the initiation of the study, study design, implementation, data collection, writing of the report and the decision to submit the report for publication.

## Introduction

### Background and rationale {6a}

Gaming has become increasingly popular since it was introduced in the 1970´s. The rapid development of video games and online gaming, especially in the last decade, has made gaming a very popular activity in broader society. In a report from Europe’s Video Games Industries 2023, 53 percent of the population aged 6 to 64 reported gaming in the last year, with a mean time of nine hours per week. The mean age was 31 years, indicating that it is not only children and adolescents participating in gaming [1]. Gaming has several positive effects, for example, increased cooperative ability, spatial perception, flexibility in attention and problem solving [2–6]. Gaming has also been used as a tool in health care, for example, to improve balance, increase physical activity through exercise games and in the treatment of post-traumatic stress disorder (PTSD) [7–11]. For most people, gaming is associated with pleasure and recreation and engaging in gaming is also positively correlated with good mental health [12, 13]. Moreover, gaming can also be used as a coping mechanism for underlying difficulties. Some individuals find social acceptance in the gaming community, which they may not otherwise experience in their daily lives [14]. Other individuals may appreciate the structure and predictability of the games, for example, in extensive multiplayer online games [15]. However, as the popularity of gaming increases, the negative effects of gaming have been more commonly observed [16].

For some individuals, gaming takes up an increasingly large part of life and eventually affects work, school and personal relationships. When the new version of the World Health Organization´s diagnostic classification system (ICD-11) was presented in 2018, gaming disorder (GD) was included as a diagnosis [17]. Gaming disorder is described as a pattern of persistent or recurrent gaming behavior (digital- or video-gaming), which may be online or offline, and is defined by the following three criteria 1) impaired control over gaming 2) increasing priority given to gaming to the extent that gaming takes precedence over other life interests and daily activities and 3) continuation or escalation of gaming despite the occurrence of negative consequences.

A few years earlier, the Diagnostic and Statistical Manual of Mental Disorders (DSM-5), presented Internet Gaming Disorder (IGD) as a potential disorder that needs to be studied more extensively [18]. It is suggested that five out of the following nine criteria should be present to meet the criteria for the disorder: 1) high pre-occupation with gaming, 2) withdrawal symptoms, 3) increased tolerance to gaming, 4) unsuccessful attempts to stop or reduce gaming, 5) loss of interest in other hobbies or activities, 6) excessive gaming despite negative consequences, 7) deception about gaming activities towards others, 8) use of gaming as escape or relief from a negative mood, and 9) jeopardized or lost relationships, jobs, or educational or career opportunities.

Gaming disorder has been associated with several different psychiatric comorbidities. For example, previous studies have shown correlations with depression and anxiety [16, 19–21], autism [22], and Attention Deficit Hyperactivity Disorder (ADHD) [23]. In addition, many individuals diagnosed with GD experience low quality of life [24].

It is still not fully known why an individual develops GD. It is likely that it can not be explained by one single factor. Király et al. suggests that three different factors could affect the development of GD: gaming related, individual and environmental factors [19]. For example, gaming companies design games to be as engaging and rewarding as possible. Further, individual risk factors may also play a role. Previous findings point to male gender, younger age and psychiatric comorbidity as risk factors for GD. Family, school and individual infrastructure for gaming can be seen as environmental factors affecting gaming habits [19]. In an influential model, Brand et al. [25] summarized research on different kinds of behavioral addictions, including GD, and developed a theoretical model showing how unproblematic behavior can develop into addiction. Specifically, the model describes how predisposing individual vulnerabilities, together with cognitive and affective responses, can cause behaviors to become addictive, and how these interactions change during the addictive process. They describe the early stages of the addictive process as characterized by gratifying experiences when engaging in the behavior, but that the behavior is controlled by active decisions to engage in it or not. In later stages, the behavior is instead more habitual and difficult to control and may also more often serve to avoid negative feelings than being a purely rewarding activity [25].

Gaming disorder can cause both personal suffering, absenteeism from school or job and societal costs, as the individual is unable to function in his or her everyday life. Losing important years in school could have major consequences later in life. Thus, it is urgent to establish an effective treatment for GD since there is currently no “gold standard”. Several methods have been studied, with varied results. One of the most studied approaches is cognitive behavioral therapy (CBT). However, the level of evidence for CBT is still low due to methodological reasons and poorly designed studies, lowering the quality and degree of evidence. In a recent meta-analysis that included 38 studies, it was concluded that CBT seems to have a good effect, but the reliability of the results may have been biased due to the inclusion of small studies with low scientific quality [26].

For GD, no evidence-based CBT program for adolescents and adults exists in Sweden. Therefore, we aim to evaluate a CBT-manual based on standardized modules which can be flexibly combined depending on individual needs.

### Objectives {7}

The primary objective of this trial is to examine the effects of a 12-week CBT-intervention on GD-symptoms, compared to a waitlist control. The secondary objective is to study the effects on weekly hours of gaming. The hypothesis is that CBT will lead to a reduction of GD-symptoms and a decrease of weekly hours in gaming, compared to the wait list control.

### Trial design {8}

The study is designed as a two-armed RCT with an allocation ratio of 1:1 to each arm. The primary endpoint is symptoms of GD at end of treatment (time point 3). We aim to investigate if the group receiving active treatment have better outcomes than the wait list control, hence the study is designed as a superiority trial.

## Methods: participants, interventions and outcomes

### Study setting {9}

This is a single-center study including participants from all geographical areas of Sweden. The trial will be performed at the Clinic for Gambling Disorder and Screen Health, Sahlgrenska University Hospital, Gothenburg, Sweden, and the treatment sessions will be held on site at the clinic or digitally via computer or smartphone. The clinic specializes in the treatment of gambling and gaming disorder.

### Eligibility criteria {10}

There are four criteria for inclusion: 1) meeting at least five out of nine diagnostic criteria for IGD according to the DSM-V; 2) 15 years of age; 3) able to read and write in Swedish; 4) provide informed consent to participate in the study. Exclusion criteria are as follows: 1) somatic or psychiatric disease that is contraindicative for treatment or that seriously limits participation in the study (e.g., ongoing psychotic, manic or hypomanic episode or neurodevelopmental disorders with severe disability); 2) an ongoing significantly elevated risk of suicide (based on assessment in a structured clinical interview); 3) participation in another ongoing psychological treatment with a similar content as in this trial, or plan to start such treatment during the time of the study; and 4) has started or changed medication for any psychiatric problem during the last month.

The intervention will be conducted by clinical psychologists with documented experience in CBT.

### Who will take informed consent? {26a}

Informed consent will be obtained by a research assistant, either digitally or on paper. The digital consent form will be signed using a secure electronic ID (Bank-ID, Finansiell ID-Teknik BID AB).

### Additional consent provisions for collection and use of participant data and biological specimens {26b}

N/a. No biological specimens will be collected in this study; therefore, no additional consent will be obtained.

## Interventions

### Explanation for the choice of comparators {6b}

Today, no evidence based “gold standard” treatment exists for GD. Therefore, there are no obvious choices of comparators. For this reason, the CBT treatment is compared to a waitlist control. Participants in the waitlist control group will, after the control period, be offered the same treatment as participants in the intervention group.

### Intervention description {11a}

Every patient that seeks treatment for GD at the clinic will be booked for an initial clinical interview with a psychologist or social worker. Before the visit, the patients will answer a number of questionnaires (see Table 2, Timeline). Included in the first interview is a semi-structured diagnostic interview about GD; sociodemographic and anamnestic information; current psychiatric status, including assessment of suicidal risk; and gaming habits. Patients meeting the inclusion criteria for the study will receive information about the trial.

After initial assessments, a research assistant will contact all patients by telephone and go through the Mini International Neuropsychiatric Interview (M.I.N.I.−7.0.1) [27]. The M.I.N.I. is a brief interview aimed at detecting psychiatric comorbidity (see Sect. 18a) and will enable further identification of reasons for exclusion. If no exclusion criteria are met, the patients will be asked about participation in the study. Patients that accept will sign the informed consent form.

Before randomization, participants will be invited to a psychosocial assessment conducted by a social worker. Topics covered in the interview will include living situation, financial situation, and social network. If any difficulty is identified and deemed to have a negative effect on the possibility of taking part in the treatment, an intervention will be initiated. For example, an individual with difficulties taking care of the household may need support from social services.

If no other interventions are needed before CBT treatment, the participants will be randomized to one of the two arms: the intervention group or the control group. For participants that need extra interventions before the treatment starts, randomization will be postponed until it is deemed appropriate to start the treatment.

#### Intervention group

##### CBT manual

The main part of the intervention is based on a CBT manual comprising nine different modules, each addressing an area identified as problematic for a majority of this patient group, for example, “Making gaming more difficult” (to address problems associated with diminished control over gaming) and “Emotions” (because gaming often serves as a dominant strategy to regulate emotions) (see Table 1). In addition to the main modules, there will also be three optional modules with content valid for some of the participants. Every participant will have an individual treatment plan, including the main and optional modules corresponding to individual needs and difficulties related to gaming. Based on clinical judgment, the length and number of modules may therefore differ between patients.

**Table 1:** Modules included in the CBT manual

Module	Content
1	Goal setting
2	Understanding your gaming
3	Planning of time
4	Making gaming more difficult. Mid-treatment assessment
5	Alternative activities
6	Procrastination
7	Emotions
8	Thoughts
9	Relapse prevention. Post-treatment assessment
Optional	Support for home assignments
Optional	In-game purchases
Optional	Handling obstacles

The sessions are planned for a duration of between 45 to 60 min. During each session, the psychologist will review last week's homework together with the participant and present a new topic. If more time is needed for last week’s topic, this will be accommodated. In between every session the participants will work on take home assignments related to the different topics.

To ensure treatment fidelity over time, the therapists will document which parts of each module have been covered in the session.

The treatment will last for approximately 12 weeks, with one session every week, and will be led by clinical psychologists with good experience in CBT. Assessments will be made at the first, fourth and last session (T1, T2 and T3, see Table 2, Timeline).

**Table 2:**
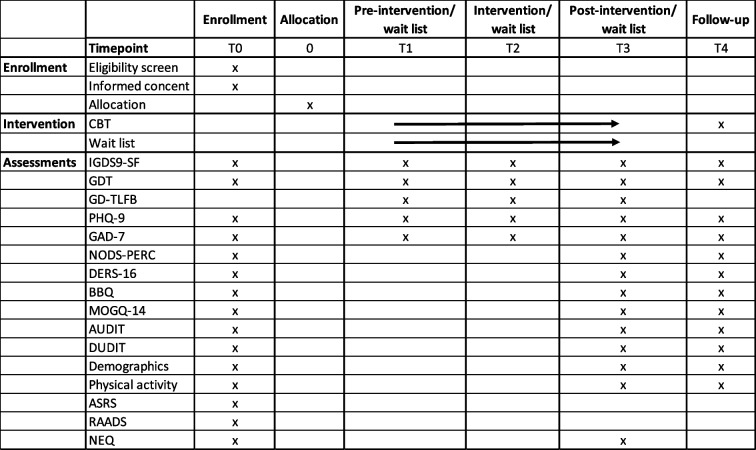
Timeline for participants included in the trial

*T* Timepoint, *CBT* Cognitive Behavioral Therapy, *IGDS9-SF* Internet Gaming Disorder Scale-Short Form, *GDT* Gaming Disorder Test, *GD-TLFB* Gaming Disorder- Time Line Follow Back, *PHQ-9* Patient Health Questionnaire -9, *GAD-7* General Anxiety Disorder-7, *NODS-PERC* National Opinion Research Center DSM-IV Screen for Gambling, *DERS-16* Difficulties in Emotion Regulation Scale, *BBQ* Brunnsviken Brief Quality of Life Scale, *MOGQ-14* Motives for Online Gaming Questionnaire, *AUDIT* Alcohol Use Disorders Identification Test, *DUDIT* Drug Use Disorders Identification Test, *ASRS* Adult ADHD Self-Report Scale, *RAADS* Ritvo Autism and Asberger Diagnostic Scale, *NEQ* Negative Effects Questionnaire

#### Family sessions

Each participant’s family and/or close friends will be offered 3–4 sessions together with the participant and a social worker, in parallel with the CBT treatment. The purpose is to set common goals, provide information on how the family can support the participant through the treatment, and give advice on how to communicate within the family.

#### Control group

Participants allocated to the control group will be put on a waitlist for approximately 12 weeks. The participants in the control group will be distributed the same questionnaires and gaming diary at the same time points as the intervention group (see Table 2, Timeline). After the waitlist period, the participants in this group will be offered the same treatment as the intervention group.

### Criteria for discontinuing or modifying allocated interventions {11b}

Some factors may lead to the discontinuation or modification of the intervention: 1) the participant wishes to withdraw from the trial, 2) the occurrence of some of the exclusion criteria, 3) incidence of serious adverse events (e.g. hospitalization, suicide attempt or significant worsening of psychiatric symptoms requiring extensive additional out-patient care). and 4) other problems are judged to be more severe than GD and more important to address (e.g. if a personal crisis occur or if it due to problems in school is judged to be more important to focus all efforts on school interventions).

### Strategies to improve adherence to interventions {11c}

Several steps have been taken to increase the participant´s ability to follow the protocol. The participants will receive a reminder by SMS before each session. At every session, the participants will also be reminded of the importance of doing the home assignments and filling out the gaming diary. Besides being an important part of the treatment, the gaming diary is also a method for monitoring adherence to the protocol. Research personnel will monitor whether the questionnaires are answered as planned and provide reminders to the therapists or participants. If needed, the participant will be encouraged to involve family or close friends in the treatment, to help with home assignments or other practical engagements. Also, the amount of time allocated to the different modules will be adjusted based on individual needs. The length and order of each module will be carefully documented in the medical journal. The participants will also be urged to contact the clinic if any problem occurs during the treatment period.

### Relevant concomitant care permitted or prohibited during the trial {11d}

Any treatment of somatic illness will be permitted during the study. However, psychological, or psychiatric treatment with a similar content as in this trial will be prohibited. Starting or changing medication for any psychiatric problem during the treatment period will be registered but not prohibited.

### Provisions for post-trial care {30}

If a participant needs extra support or care after the CBT treatment it will be provided at the clinic or the participant will be referred to the appropriate treatment provider. During the telephone follow-up (3 months after end of treatment) it will be possible to identify patients that may have a relapse in GD and provide further care. After the active treatment period, the participants will be free to seek any form of care they need.

All participants will be covered by a patient insurance and will be able to seek compensation if they have suffered harm from treatment.

### Outcomes {12}

#### Primary

The primary outcome of this study is symptoms of GD. To measure symptoms of GD, a Swedish version of the Internet Gaming Disorder Scale 9—Short Form (IGDS9-SF) will be used. This is one of the most frequently used questionnaires internationally for measuring symptoms related to excessive gaming [28]. It is based on the nine criteria for IGD in the DSM-5. The primary endpoint will be the mean change in IGDS9-SF from baseline to end of treatment and will be distributed at all time points for data collection (see Table 2, Timeline).

#### Secondary

Weekly hours of gaming will be monitored during the treatment and control period, using the Gaming Disorder Timeline Follow-Back (GD-TLFB). The mean change from the start of treatment to end of treatment will constitute the outcome for GD-TLFB. Gaming time is not directly connected to GD [29], but spending excessive amounts of time gaming has been identified as a risk factor for GD [30].

To complement the diagnostic criteria in the DSM-5, a shorter questionnaire based on ICD-11, the Gaming Disorder Test (GDT), is included in the test battery, [31, 32]. The mean change from baseline to end of treatment will constitute the outcome for GDT, that will be distributed at all time points for data collection (see Table 2, Timeline).

#### Exploratory

The occurrence of, and possible effect of the intervention on comorbidity of depression and anxiety will be used as exploratory outcomes of this trial. These conditions will be assessed using the Patient Health Questionnaire-9 (PHQ-9) [33] and General Anxiety Disorder 7-Item Scale (GAD-7) [34]. Previous research has shown that both depression and anxiety are common in patients with GD [16, 19–21]. Data for depression and anxiety will be collected at every time point for assessment (see Table 2, timeline), and data will be aggregated as the mean change from baseline to follow-up.

We will also screen the participants for gambling problems using the National Opinion Research Center DSM-IV Screen for Gambling (NODS-PERC) at time points 0, 3 and 4. Emotional regulation will be assessed by the Difficulties in Emotion Regulation Scale (DERS-16) at time points 0, 3 and 4.

Another area of interest is quality of life. Data from our pilot study showed low levels of quality of life among patients with GD [24]. In order to determine the treatment's effect on quality of life, the Brunnsviken Brief Quality of Life Scale (BBQ) [35] will be used. This questionnaire will be included in the data collection at time points 0, 3 and 4. Mean change from baseline to follow up will be used as outcomes for the BBQ. In addition to questionnaires concerning psychological health, the participants will also answer several gaming related questions, for example, main type of game or if the participant plays alone or with others.

It has also been seen that individuals with GD seem to have a low level of physical activity [36], which is a risk factor for poor physical and psychological health [37, 38]. We want to study the level of physical activity at baseline and investigate whether a decrease in gaming time increases the time spent engaged in physical activity. At time points 0, 3 and 4, the participants will answer questions regarding physical activity level and sedentary time.

### Participant timeline {13}

#### Sample size {14}

The power calculation is based on an expected reduction in the symptoms of GD, measured by IGDS9-SF. While the IGDS9-SF demonstrates robust psychometric properties [28, 39], we have not found any clinical studies using this instrument as an outcome measure after intervention. However, in our pilot study [24] we included a set of items highly resembling the IGDS9-SF and observed a mean decrease of 10.9 (SD 10.6) points. There was no wait list control in the pilot study, but there was a waiting time between first visit and start of treatment. During this time, we saw a small mean decrease of 0.5 on the same symptom measure. Based on this, we would not expect any or a very small reduction in the wait list control group. However, in the RCT the control group will receive questionnaires including questions about time spent gaming. This kind of self-monitoring might lead to some additional changes in behavior that would occur while waiting for treatment.

Based on our pilot data [24], we have therefore concluded that a reasonable assumption is a 10-point reduction in the intervention group and a 5-point reduction in the control group. Hence, we calculated the sample size to be able to detect a difference of 5 points between the groups. With a standard deviation of 10, power of 80 percent and significance level of 5 percent, the required group size is 64 individuals per arm or 128 individuals in total. Assuming a drop-out rate of 20 percent, approximately 160 individuals would need to be recruited.

A 5-point difference on the IGDS9-SF scale corresponds to changes in frequency of at least two symptoms of GD. It is also equivalent to the minimal clinically important difference defined as half the standard deviation of the change scores[40]. Due to the lack of clinical evidence using this particular questionnaire, we have also made parallel calculations of sample size using data about time spent gaming. The sample size (128 individuals) would allow for detection of about an eight hour decrease in time spent gaming per week, i.e. a full workday. Based on clinical judgment and data from our pilot study, we have therefore made the decision that this sample size would allow for detection of clinically meaningful changes.

Power was calculated based on an independent samples t-test for two groups, that is, the Student’s t-test. Calculations were performed using the pwr.t.test from the pwr r-package.

### Recruitment {15}

Information about the study and contact information for the clinic will be distributed through the clinic’s website and in waiting rooms in primary care and psychiatric facilities. Also, information will be spread through social media (e.g., Facebook) and platforms for gaming (e.g., Discord). Patients seeking treatment for GD at the clinic for Gambling Addiction and Screen Health will be screened for eligibility and informed about the study by the health care personnel performing the initial interview.

As estimated by the power calculation, 160 participants will be recruited over approximately three years from first inclusion. The recruitment process will be closely monitored, and each inclusion will be reported to study personnel within one week.

## Assignment of interventions: allocation

### Sequence generation {16a}

The participants will be randomized using simple, computer-generated randomization. The allocation ratio will be 1:1 to each arm. No stratification or blocking procedures will be applied.

### Concealment mechanism {16b}

A computer-generated random numbers randomization was done, using the Clinical Randomization Tool (Clinical Trial Randomization Tool. The National Cancer Institute's Division of Cancer Prevention, https://ctrandomization.cancer.gov. Accessed September 13, 2023). Only one person will have access to the randomization list, which will be stored on a safe server.

Sealed, opaque envelopes will be used in the implementation of the allocation sequence. To conceal the sequence until interventions are assigned, the envelopes will be numbered and contain a note with “Intervention” or “Control”, according to the list from the randomization procedure. Thus, the possibility of influencing the randomization is limited. The envelope for each participant will not be opened until all baseline measures and preparatory interventions are completed.

### Implementation {16c}

The research personnel will generate the allocation sequence and distribute the sealed envelopes. The enrolment of participants will be implemented by psychologists in weekly treatment conferences. The research personnel will also register the results of the enrolment and assign participants an individual ID number. The ID-number will be used in all registers and study files.

## Assignment of interventions: blinding

### Who will be blinded {17a}

The possibilities for blinding in this trial are limited. The health care personnel providing the treatment (psychologists and social worker) cannot be blinded, nor can the research personnel who handle all data. The only personnel that will be blinded is the statistician, who will receive a coded data file.

### Procedure for unblinding if needed {17b}

Not applicable.

## Data collection and management

### Plans for assessment and collection of outcomes {18a}

Several self-report questionnaires and semi-structured diagnostic instruments will be used in this trial.

Data will be collected digitally at several time-points before, during and after the intervention (see Table 2 Timeline). For patients who are not able to access the digital questionnaire service, it will be possible to fill out the questionnaires on paper.

The first assessment will be done before the initial visit to the clinic (T0). The second will occur right before the start of treatment (T1). Measurements will continue through the treatment period with questionnaires distributed at session four (T2) and nine (end of treatment) (T3). If the treatment takes longer than expected an extra measurement will occur during the treatment period.

The participants will be followed up by telephone three months after the end of treatment. In a subsequent study, the participants will also be followed up after 6, 12, 18 and 24 months, with the same questionnaires as in the study described in this protocol.

The primary outcome measure is:

#### Internet Gaming Disorder Scale- Short Form (IGDS9-SF)

When measuring GD, this is one of the most frequently used questionnaires internationally [28]. It is based on the nine criteria for IGD in the DSM-5. There is one question for every criterion, answered on a five-point Likert scale representing “never”, “rarely”, “sometimes”, “often” and “very often”. The score ranges from 9 to 45 (higher scores reflect more problems related to gaming), with a suggested clinical cut-off at or above 32 [41]. At least five of the questions answered with “very often” indicates that the criteria for a diagnosis of IGD have been met [28]. The scale is not a diagnostic tool, but an instrument to assess severity of GD. IGDS9-SF has been validated against weekly gameplay and the IGD-20-test [42] showing good validity and a Cronbach´s alpha of 0.87. In this study, the total score is used as a measure of severity of IGD and will be included at all time points from the first visit (T0) to the follow-up after 3 months (T4). A lower score post-treatment compared to pre-treatment is seen as a positive outcome. For this study, an adapted version will be used, measuring symptoms during the last four weeks.

The secondary outcomes are:

#### Gaming Disorder Test (GDT)

This four-item questionnaire has been developed from the ICD-11 diagnostic criteria of GD [32]. Answers to the questions are given on a 5-point Likert scale ranging from 1 to 5, representing “never”, “rarely”, “sometimes”, “often” and “very often”. The score ranges from 4 to 20, with higher scores indicating a higher degree of gaming problems. To score “4” or “5” on a question indicates that the criterion is met. As with IGDS9-SF, GDT is not a diagnostic tool, but an instrument to assess severity of GD. The Gaming Disorder Test has been validated against IGDS9-SF (r = 0.82) and has a Cronbach´s alpha of 0.84. For this study an adapted version will be used, measuring symptoms during the last four weeks.

#### Gaming Disorder Timeline Follow-Back (GD-TLFB)

This gaming-related diary is the third main outcome measure. It was originally developed to track alcohol-consumption and has been adapted to measure gaming behavior [43]. The adapted version collects measurements on a weekly basis: number of days gaming, total hours of gaming and leisure time without screen (e.g., reading a book, meeting friends, or exercising). The diary will be filled out weekly during treatment to evaluate the process. It will also be used as a visual tool for the participants to gain an overview of their gaming habits. The control group will also fill out the gaming diary during the control period.

Exploratory outcomes are:

#### Patient Health Questionnaire-9 (PHQ-9)

This questionnaire consists of nine items screening for symptoms of depression during the last two weeks [33]. The four response alternatives are “not at all”, “several days”, “more than half of the days” and “nearly every day” with a possible total score ranging from 0 to 27. The PHQ-9 has been developed according to diagnostic criteria in the DSM-4, and the score can be used to assess the severity of depressive symptoms. The internal reliability of PHQ-9 has been shown to be good, with a Cronbach´s alpha of 0.89. Validity was tested against the Medical Outcomes Study Short-Form General Health Survey (SF-20), and the results showed high validity. Based on the total score the level of severity is classified as none (0–4), mild (5–9), moderate (10–14), moderately severe (15–19) or severe (20–27). PHQ-9 has been shown to have high validity in detecting the severity of depression. The total score will be used in the analyses to track variations throughout the study. Assessments will be made at every time point (T0-T4).

#### General Anxiety Disorder 7-Item Scale (GAD-7)

The General Anxiety Disorder-7-Item Scale was developed as an instrument to measure the presence and severity of symptoms of anxiety [34]. It is a seven-item questionnaire screening for symptoms during the last two weeks. The total score is 21, with cut-off points at 5, 10 and 15, indicating minimal (0–4), mild (5–9), moderate (10–14) and severe (15–21) levels of anxiety. The response categories for each question are “not at all”, “several days”, “more than half the days” and “nearly every day”. Both internal validity (Cronbach´s alpha 0.92) and test–retest reliability (0.83) have been shown to be good. The total score will be assessed at time points 0 to 4.

#### National Opinion Research Center DSM-IV Screen for Gambling (NODS-PERC)

This is a short version of the original 17-item scale NODS. It was developed based on four of the DSM-4 criteria for gambling disorder: preoccupation, escape, chasing losses and social consequences. It is used as a screening tool for gambling problems and contains four questions regarding difficulties related to gambling. The questions are answered with “yes” or “no”. If at least one question is answered as “yes”, gambling problems may be present [44].

#### Difficulties in Emotion Regulation Scale (DERS-16)

This is a self-rating scale developed to measure the level of difficulties in emotion regulation. Answers are given on a five-point Likert scale ranging from 1) almost never to 5) almost always. The total score ranges from 16–80, where higher scores indicate higher levels of difficulties in emotion regulation. It is built on five subscales: clarity, goals, impulse, strategies and non-acceptance. Test–retest reliability is 0.85, and Cronbach´s alpha is between 0.92 and 0.95 [45, 46].

#### Brunnsviken Brief Quality of Life Scale (BBQ)

The Brunnsviken Brief Quality of Life Scale was developed in Sweden to measure an individual´s subjective quality of life in a clinical setting [35]. The questionnaire is divided into six different life areas such as “view on life”, “creativity” and “friends and friendship”. Each life area is represented by two statements: the first applies to “satisfaction” and the second applies to “importance”. Both statements are answered on a five-step Likert scale, scored visually from 0–4, with 0 representing “do not agree at all” and 4 “agree completely”. The score on the “satisfaction” statement is multiplied with the “importance” statement. Thus, the total possible score is 96, with higher scores indicating higher perceived quality of life, and with a suggested clinical cut-off score at 52.5. The Cronbach´s alpha was 0.76, and the internal validity was shown to be 0.82. The BBQ will be included in the initial measurements (T0), post-treatment (T3) and at the 3-month follow-up (T4).

#### Motives for online gaming questionnaire (MOGQ-14)

The Motives for Online Gaming Questionnaire is a self-report instrument measuring motives for gaming [47]. It covers seven different motivational factors: social, escape, competition, coping, skill development, fantasy and recreation. The questions are answered on a five-point Likert scale ranging from 1 (almost never) to 5 (most of the time). The questionnaire can be used for various types of games and has been validated in a Swedish context [48].

#### Alcohol Use Disorders Identification Test (AUDIT)

The Alcohol Use Disorders Identification Test is a screening tool for alcohol related problems and identifies individuals with harmful use of alcohol [49]. It consists of ten items divided into three areas: alcohol consumption (item 1–3), symptoms of dependence (item 4–6) and negative consequences of alcohol consumption (item 7–10). Items 1–8 have five response alternatives, scored 0 to 4, while items 9 and 10 has three alternatives, scored 0, 2 and 4. The maximum score is 40, with a cut-off score of 6 for women and 8 for men, indicating hazardous or harmful drinking. The Cronbach´s alpha was 0.82, and test–retest reliability was 0.93. This questionnaire will be administered at the initial visit (T0), post-treatment (T3) and at follow-up (T4).

#### Drug Use Disorders Identification Test (DUDIT)

The Drug Use Disorers Identification Test is a screening tool for the use of drugs and events of drug-related consequences [50]. It is an 11-item instrument categorized in the three areas drug use, dependence symptoms and negative consequences of drug use [50]. Items 1 to 9 have five response alternatives scored 0 to 4, and items 10 and 11 has three alternatives scored 0, 2 and 4. It has a total possible score of 44. Clinical cut-offs for problematic use are 1 for women and 3 for men, and the Cronbach´s alpha was 0.80. This questionnaire will be distributed at the initial visit (T0), post-treatment (T3) and follow-up (T4).

#### Physical activity and sedentary behaviour

The level of physical activity and sedentary behavior will be evaluated using three questions developed by the Research Group for Physical activity and Health at the Swedish School of Sport and Health Sciences. These questions are validated against accelerometer, objective tests (submaximal oxygen uptake test, balance test and vertical jump test) and cardiovascular risk factors. One question measures time spent in exercise training (six response alternatives ranging from 0 to more than 120 min per week), while another question measures time spent in everyday physical activity (seven response alternatives ranging from 0 to more than 300 min per week). The last question measures time spent sitting during a day (seven response alternatives ranging from “almost all day” to “never”) [51]. These questions are recommended by the Swedish National Board of Health and Welfare.

#### Demographics and gaming-related questions

Information about education, living situation, occupation, medication, gaming-related somatic symptoms, length and height is obtained, as well as information about gaming habits. The participants are, for example, asked about preferred game genres, main type of platform used for gaming and treatment goals for gaming.

Scales used only at baseline (T0):

#### Adult ADHD Self-Report Scale (ASRS V1.1) Screener

The Adult Self-Report Scale V1.1 Screener is a shorter version of the ASRS V1.1 Symptoms Checklist and is used to identify adult individuals with symptoms of ADHD [52]. It is a six-item instrument, which asks questions about symptoms of ADHD during the last six months based on symptoms described in the DSM-4. The instrument uses a five-point response scale (0–4) representing “never”, “rarely”, “sometimes”, “often” and “very often” with a total score ranging from 0 to 24. A cut-off of 14 or above is suggested as positive for ADHD (0—9: low negative; 10—13: high negative; 14—17: low positive; 18—24: high positive). In a study by Kim et al. (2013) the Cronbach´s alpha was shown to be 0.89 and the test–retest reliability was 0.88 [53]. Although it was developed for an adult population, the ASRS screener has also shown good reliability for use in adolescents [54].

#### Ritvo Autism and Asberger Diagnostic Scale (RAADS-14 Screen)

The Ritvo Autism and Asberger Diagnostic Scale is a self-assessment form screening for autism spectrum syndromes in adults [55]. The scale has three domains: mentalizing deficits, sensory reactivity, and social anxiety. The response alternatives are: 1) true now and when I was young, 2) true only now, 3) true only when I was young, and 4) never true. The maximum score is 42, with a cut-off of 14 indicating symptoms of autism spectrum disorder. The Cronbach´s alpha was above 0.8.

Scale used only during treatment (T2):

#### Negative Effects Questionnaire (NEQ)

The purpose of the NEQ is to measure negative or adverse effects of psychological treatment, as experienced by the patient [56]. It contains 20 statements connected to experiences from the treatment, for example, “I felt more worried” and “Unpleasant memories resurfaced”. The statements are answered by “yes” or “no”. If the answer is yes, the statement is graded on a five-point Likert scale with the response alternatives 0) “not at all”, 1) “slightly”, 2) “moderately”, 3) “very” or 4) “extremely”. The respondent then reports whether the experience was a result of the treatment or other circumstances. The total score ranges from 0 to 80, and can be divided into five factors: symptoms, quality, stigma, dependency and hopelessness. The reliability has been shown to be good, with a Cronbach´s alpha of 0.95 [57].

Clinical interviews used at screening:

#### Semi-structured diagnostic interview for gaming disorder

This clinical interview will be used as a support for diagnosing GD. It is based on the nine criteria for IGD presented in the DSM-V and is a modified version of a semi-structured interview developed by Vadlin et al. [58]. The proposed cut-off for diagnosis is fulfilment of five or more criteria.

#### The Mini- International Neuropsychiatric Interview (MINI-7.0.1)

The Mini International Neuropsychiatric Interview is a structured screening interview for common psychiatric disorders. It has been updated to reflect the changes in the DSM-5 [27, 30]. It includes questions assessing a wide range of psychiatric disorders including mood and anxiety disorders, suicide risk, and use of psychoactive substances. It also includes items assessing ADHD. The Swedish version of this structured clinical interview will be used to assess psychiatric comorbidity in the assessment of exclusion criteria [59]. It has shown excellent inter-rater reliability (all kappa values over 0.75) and good test–retest reliability (61% of kappa values over 0.75) [27].

### Plans to promote participant retention and complete follow-up {18b}

To encourage the participants to complete the follow-ups, they will be rewarded with a gift card after the post-treatment assessment (T4). Reminders will be sent out by SMS before each follow-up. If the participant discontinues participation before end of study for any reason, they will be contacted by telephone. Standardized questions will be asked in order to complete questionnaires equivalent to those in T3 (see Table 2) as well as the reasons for discontinuation (optional).

### Data management {19}

New participants will be registered and coded every week. The collected data will be regularly entered in IBM SPSS (once a month). All digital data will be stored on a safe server only accessible by the involved researchers through personal identification. Data on paper will be stored for at least 25 years. Data quality will be promoted through the automatic scoring of questionnaires. Range checks for data values will also be performed.

### Confidentiality {27}

All information collected on paper will be stored in a safe placed in a locked room, with the key stored in a locker that can only be accessed with a code. The digital questionnaires will be downloaded to a secure server that can only be accessed by personnel involved in the trial. Personal data in the dataset will be coded and it will not be possible to identify individuals. The key code will be stored separately in a password protected digital folder. All data will be anonymized.

### Plans for collection, laboratory evaluation and storage of biological specimens for genetic or molecular analysis in this trial/future use {33}

Not applicable. No biological specimens will be used in this study.

## Statistical methods

### Statistical methods for primary and secondary outcomes {20a}

Descriptive data will be presented by treatment arm and, where applicable, by timepoint. For numeric variables, summary statistics will include the number of available and missing measurements, mean, standard deviation (SD), median and minimum and maximum values. Treatment comparisons for primary, secondary and exploratory endpoints will be conducted using analysis of covariance (ANCOVA), adjusted for baseline values.

The primary efficacy analysis, which assesses the change in IGDS9-SF from baseline to 12 weeks between the CBT and control groups, will be conducted using analysis of covariance, adjusted for baseline values, with missing data handled through multiple imputation using predictive mean matching. The treatment effect will be tested at a significance level of α = 0.05. Results will be reported as the mean difference with 95% confidence intervals.

Secondary efficacy analyses of changes in GDT and GD-TLFB from baseline to 12 weeks between the CBT and control groups will be conducted using the same methodology as the primary efficacy analysis. To control the overall Type I error rate for secondary endpoints, an alpha-splitting testing procedure will be employed. The two secondary endpoints—GD-TLFB weekly hours and changes in GDT scores from baseline to 12 weeks—will initially be tested with an alpha level of 0.025 each. If the first test (i.e., GD-TLFB) demonstrates statistical significance, the entire probability mass will be transferred to the next secondary endpoint (i.e., GDT), which will subsequently be tested at the 0.05 significance level, with results reported as the mean difference and corresponding 95% confidence intervals.

All statistical tests will be two-tailed, with results presented as mean differences and accompanied with corresponding 95% confidence intervals.

### Interim analyses {21b}

We plan to conduct interim analyses when approximately 50% of the planned total sample has finished the trial period [60]. These analyses will be made to investigate the actual standard deviation in the study sample (as previous knowledge about this is extremely limited), to make a more informed decision about sample size. The interim analyses also enable decisions about implementation in regular care as fast as possible, as treatment options for this group of patients are lacking. Based on the data at that point of time sample size will be adjusted. These decisions will be made by the trial steering committee in collaboration with the assigned statistician.

### Methods for additional analyses (e.g., subgroup analyses) {20b}

There are no sub-group analyses planned.

### Methods in analysis to handle protocol non-adherence and any statistical methods to handle missing data {20c}

In this trail, protocol adherence will be defined as attending at least four CBT sessions and responding to at least one post-baseline measurement (T2-3). The therapist will document each session in the medical journal.

Missing data will be addressed through multiple imputation by chained equations (fully conditional specification) with predictive mean matching. The imputation model will include auxiliary information from measurements of the efficacy variable of interest collected at previous and subsequent visits. A total of 50 imputed data sets will be generated, and the results pooled according to Rubin’s rules.

### Plans to give access to the full protocol, participant level-data and statistical code {31c}

The data sets used and/or analyzed during the current study can be made available by the corresponding author upon reasonable request.

## Oversight and monitoring

### Composition of the coordinating center and trial steering committee {5d}

To handle day-to-day trial conduct, a trial management group is organised, composed of two researchers connected to the clinic. They attend the weekly clinical meetings together with the therapists. The purpose of these meetings is to review participant progress and clinical concerns (e.g. AEs), ensure consistency in the CBT-treatment and adherence to the protocol, and address problems related to the implementation of the treatment. These meetings contribute to the overall trial quality and support effective team coordination. Clinicians participating in the trial will be responsible for participant recruitment, delivery of the intervention and data collection.

There is also a trial steering committee that will meet at regular intervals and is responsible for monitoring the progress of the trial, ensuring compliance with the protocol, reviewing information from related studies, communicating updates to relevant stakeholders, and advising on the publicity and presentation of all aspects of the trial.

### Composition of the data monitoring committee, its role and reporting structure {21a}

N/a. The present study is judged as a low-risk trial, not involving any medications or medical equipment.

### Adverse event reporting and harms {22}

In this trial, adverse events (AEs) refer to any untoward occurrence in a study participant, regardless of its relationship to the intervention (e.g. worsening of psychiatric symptoms). Serious adverse events refer to e.g. hospitalization, suicide attempts or significant worsening of psychiatric symptoms requiring extensive additional out-patient care.

The therapists meet the participants once every week and will have adequate opportunities to detect AEs. The questionnaires about psychiatric symptoms during treatment will also be used to collect information about AEs, such as worsening of psychiatric symptoms. The Negative Effects Questionnaire used at the end of treatment will also assist in identifying AEs, as well as the follow-up assessments at three months after treatment. Adverse event data from questionnaires will be systematically collected by a designated member of the research team at each scheduled assessment.

Adverse events identified by the therapist or spontaneously reported will be registered in the eCRF of the trial dataset and reported during the weekly clinical meetings. The severity of the adverse event will be evaluated by the trial management group. The management will depend on the level and nature of the adverse event. In occurrence of an AE or SAE, the participant will be referred to relevant care. Further, SAEs will be registered in the internal reporting system at the hospital and reviewed by a psychiatrist who makes decisions about if the event should be reported and investigated by the national Health and Social Care Inspectorate. Serious adverse events will also be reported to the ethical review board. All collected adverse event data will include onset, duration, severity, relationship to the intervention, expectedness and outcome in accordance with the CONSORT harms statement 2022 [61].

### Frequency and plans for auditing trial conduct {23}

This is a single center trial, and no auditing is planned during the study. The research team will have regular meetings to discuss questions concerning participants, ethics and methodology.

### Plans for communicating important protocol amendments to relevant parties (e.g., trial participants, ethical committees) {25}

Adjustments of the protocol will be made in collaboration with the involved clinicians. All adjustments will be recorded in a logbook that will be readily available to all clinicians and investigators. Major changes will be reported to the ethical committee and changed in the trial registration.

### Dissemination plans {31a}

Results from this trial will be reported primarily in relevant scientific journals, reports and international and national conferences. The results will also be made available to the public via press releases and information directed to health care staff and other professionals.

## Discussion

The aim of the present study is to evaluate the effect of CBT on symptoms of GD. Cognitive behavioral therapy involves managing problematic thoughts, feelings and behaviors in more functional ways and thereby reducing symptoms. Cognitive behavioral therapy has previously been reported to have good effects in the treatment of other behavioral addictions (e.g., gambling disorder) [62]. Data from our pilot study, which included 20 participants who participated in at least four sessions using the CBT manual described in this protocol, showed a significant decrease in symptoms of GD, as well as a large reduction in weekly hours of gaming [24]. Due to these promising results, we will now implement the manual in a high-quality RCT with a waitlist control and a three-month follow-up.

The trial is carefully planned, taking into account many possible pitfalls. However, the present study has some limitations: First, and most important, the time for completion of the study is highly dependent on the rate of referral to the clinic. A regular inflow of participants is crucial to keep the timeline intact. Also, participants randomized to the control group will have to wait 12 weeks before starting the treatment, with a risk of increased impairments during the waitlist period. The choice of a waitlist control condition could also be considered a limitation but has been chosen as there is no better option or gold standard treatment to use as a comparison.

The study also has several practical implications and strengths. It is a clinical study, implemented at an established clinic that specializes in the treatment of gambling and gaming disorders. The inclusion criteria mirror the typical patients seeking treatment for GD, as psychiatric co-morbidity is common in this group. The inclusion of patients with psychiatric co-morbidity makes the results from the study applicable in clinical practice. To enable inclusion of patients with different levels of functioning, the study design allows adjustments based on individual needs, while still using a structured and well-described manualized intervention. Given the worldwide increase in gaming [63] there will be a need for treatment of GD in the health care sector, as well as increased awareness and preventive strategies in non-medical settings that encounter at-risk individuals. This trial will contribute valuable information about patients with GD and treatment outcomes.

## Trial status

Recruitment will start in August 2025, thus randomizing the first participant in September 2025. Recruitment will be completed in December 2028. This is the first version of the protocol.

## Data Availability

The final dataset will be accessible for the main researchers in this project.

## Abbreviations

GD: Gaming disorder
IGD: Internet gaming disorder
CBT: Cognitive behavioral therapy
RCT: Randomized controlled trial
IGDS9-SF: Internet Gaming Disorder Scale- Short Form
GDT: Gaming Disorder Test
GD-TLFB: Gaming Disorder Timeline Follow-Back
AE: Adverse event
SAE: Serious adverse event
